# Light People: Professor Lan Fu

**DOI:** 10.1038/s41377-023-01325-w

**Published:** 2023-12-06

**Authors:** Hui Wang, Cun Yu

**Affiliations:** Changchun Institute of Optics, Fine Mechanics and Physics, Chinese Academy of Sciences, Changchun, 130033 China

**Keywords:** Optoelectronic devices and components, Quantum dots

## Abstract

III-V semiconductors are compound semiconductor materials formed by combining group-III and group-V elements. With properties such as direct bandgap, high electron mobility, good homogeneity of large-size crystals and good lattice matching, they are widely used in micro- and opto-electronics, integrated circuits, laser communications, etc., and promise great potentials. Our Light People is someone who has long been engaged in the research of III-V semiconductor materials, structures and devices. She is Prof. Lan Fu from the Australian National University (ANU).

As an outstanding student of Prof. Chennupati Jagadish, President of the Australian Academy of Sciences, Prof. Fu is not only a leading researcher in her field, but also a scientist with an independent spirit. She is currently the Head of the Department of Electronic Materials Engineering, Research School of Physics, The Australian National University, where she leads a team in the research of III-V semiconductors.

A sincere and humble person, Prof. Fu focuses on nurturing professional attitude, critical thinking and problem solving skills in her students. In addition, she is actively involved in various professional activities, which allows her to integrate research and practice. She believes that integrity, passion, curiosity and perseverance are the key characters of a good scientific researcher and encourages her students to avoid being arrogant or rush but concentrate on good rigorous work.

For this issue of Light People, we will learn about Professor Lan Fu’s unique charisma as an academic and her fascinating life philosophy.

## Abstract (web summary)

Prof. Lan Fu from the Australian National University: Low dimensional III-V semiconductor materials, structures and devices.

## Graphical abstract

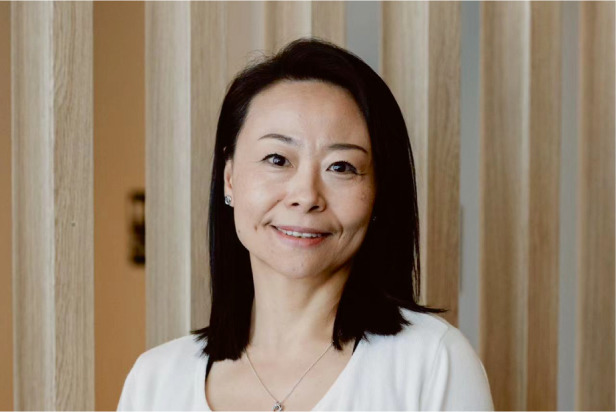

**Figure:**
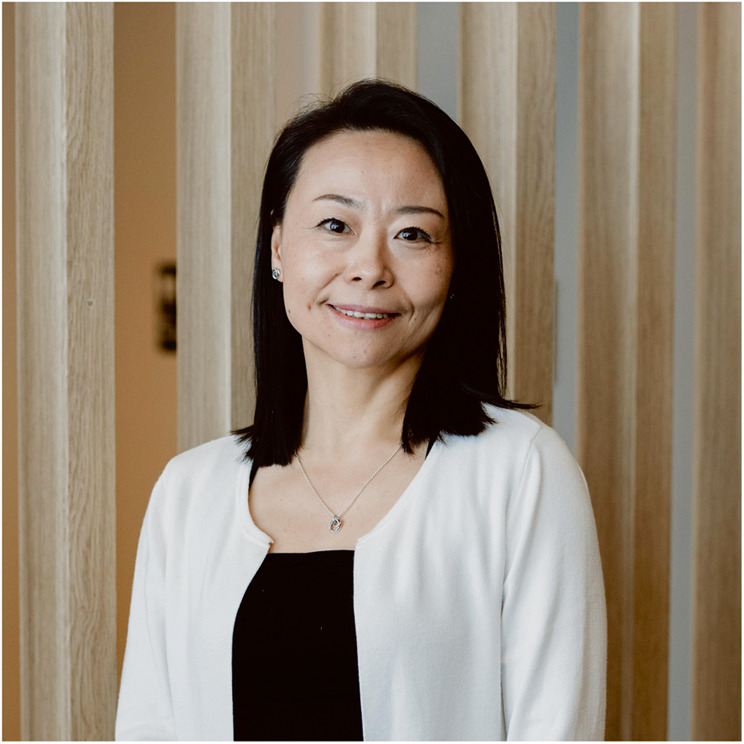

Lan Fu is a Professor and Head of the Department of the Electronic Materials Engineering at the Research School of Physics, the Australian National University (ANU). Lan Fu’s main research interests include design, fabrication and integration of optoelectronic devices (LEDs, lasers, photodetectors and solar cells) and chemical sensors, based on low-dimensional III-V compound semiconductor structures including quantum wells, self-assembled quantum dots and nanowires grown by metal-organic chemical vapour deposition (MOCVD). Prof. Lan Fu was the recipient of the IEEE Photonic Society Graduate Student Fellowship and Distinguished Lecturer Award, Australian Research Council Postdoctoral Fellowship, ARF/QEII Fellowship and Future Fellowship. She is the current Chair of IEEE Nanotechnology Council Chapters & Regional Activities Committee, Associate Editor of IEEE Photonics Journal, Beilstein Journal of Nanotechnology, and member of Editorial Board of Opto-Electronic Advances. She is also the current vice-Chair of the Australian Academy of Science National Committee on Materials Science and Engineering, and vice-President of the Australian Materials Research Society (AMRS). She has been recognised by Cosmos Magzine as one of the “50 Women of the Cutting edge of science in Australia” in 2023.

**1. Could you briefly introduce your current research focus and main work?**

Prof. Lan Fu: Our group has been working in the field of low dimensional structure based III-V semiconductor materials, structures and devices, such as quantum wells, quantum dots and nanowires. Currently, my research has been mainly focused on design, synthesis and fabrication of nanowire array based materials and structures grown by selective area metalorganic chemical vapour deposition technique, and their applications as LEDs/lasers at eye safe wavelengths, highly sensitive photodetectors for LIDAR, optical and quantum communication applications, as well as their further integration with metasurfaces to achieve highly functional and compact photonic systems. We also expand our nanowire research to the development of multiplexing wearable chemiresistive gas sensors for health and environmental monitoring and future Internet of Things (IoTs).

**2. For semiconductor devices, miniaturization, low energy consumption and intelligence are important development trends. Compared with traditional bulk semiconductor materials, what are the advantages of semiconductor nanowires?**

Prof. Lan Fu: What makes semiconductor nanowire so special is their unique quasi-one-dimensional geometry and nanoscale size, which leads to a range of interesting optical, electrical, mechanical and electrochemical properties that are different from the traditional bulk or thin film semiconductor materials. For example, due to the wavelength scale size of the nanowire structures, their light-matter interactions are very different from those of the bulk materials, leading to strong size dependent light absorption and emission properties. With their quasi-one-dimensional geometry, they are more effective in strain relaxation during epitaxial growth, easing the limitation of lattice mismatch compared with the growth of planar structures, and thus providing wider and better materials for the creation of heterostructures and/or on foreign substrates such as silicon. This, combined with the ability of their growth in both axial and radial directions, offering great flexibility for novel device designs to overcome performance trade-offs often encountered by planar structures. Furthermore, the large surface-to-volume ratio of the NWs make them promising for applications beyond the conventional III-V semiconductor based optoelectronics, such as optical and chemiresistive sensors, and photochemical devices for artificial photosynthesis. Finally, by infiltrating of the free-standing nanowires with polymer materials, the NW arrays can be peeled or lifted off from the substrate, for fabrication of flexible devices and substrate re-use. All these properties provide enormous opportunities for us to explore new fundamental physics and device technologies to achieve miniaturized, low energy consumption and intelligent semiconductor devices and systems.

**3. The concept of “metaverse” is trending now, and some colleges and universities have even set up metaverse majors. You have introduced to us how to achieve highly uniform InGaAs/InP selective epitaxy growth of multiple quantum well nanowire arrays based on metal organic chemical vapor deposition (MOCVD) through crystal plane growth control, and demonstrated high-speed micro-LED and photodetector array devices in the** tele**communication band, which can be used to produce the next generation highly integrated photonic/optoelectronic systems and highly anticipated metaverses. Could you briefly tell us the latest developments in this research?**

Prof. Lan Fu: Based on Wikipedia, a “metaverse” is a network of 3D virtual worlds connected by the use of virtual reality (VR) and augmented reality (AR) equipment, by creating immersive multi-dimensional human-to-human interactions as well as their interactions with the physical environment, where the real world is overlaid with digital fields and objects. More broadly, I think the word “metaverse” represents a much anticipated new era of industry revolution with the development of a suite of emerging technologies featuring automation, large scale communication, artificial intelligence (AI) and the IoTs, that are going to transform our life and society, just like the computer and internet. Among the various enabling technologies for the development of “metaverse”, highly integrated optoelectronic systems with the function of generation, manipulation and detection of light are indispensible. As just mentioned, III-V semiconductor nanowires offer great potential for optoelectronic device integration. Our group has been working on the development of a range of nanowire based devices and their integration with metasurfaces, including LEDs, lasers, photodetectors, solar cells and sensors. For example, one of our recently published research works is the growth and fabrication of highly uniform core-shell InGaAs/InP quantum well nanowire array based infrared micro-LEDs. We have demonstrated micro-LED arrays with small-pixel size, multiwavelength, and GHz level operation at telecommunication window, indicating good potential for the development of miniaturized, multiwavelength, ultrafast light sources for advanced on-chip optical communication systems. We are now working on design and integration of metasurfaces onto the micro-LED for optical sensing and communication.

**4. So far, what achievement of yours do you value the most, or are most proud of?**

Prof. Lan Fu: I value all the research projects that I have worked on and collaborated with. However, so far I have been mainly working on fundamental aspects of the research to publish papers. Ultimately I would be really proud if one or two of our research work can be translated into real products to improve our lives. I am very excited about the new research we have recently moved towards development of nanowire array based gas sensors. As you know, III-V semiconductors have been widely used for optoelectronic applications, but not much for chemical sensing. By leveraging our experience on nanowire based optoelectronic device design and fabrication, we have shown that III-V semiconductor nanowires presents a promising battery-free, highly integrable and compatible sensing platform for applications such as environmental and health monitoring. We have recently demonstrated high sensitivity, selectivity, self-powered, room temperature photovoltaic NO_2_ sensors that can be used for dynamic environmental monitoring. We have also demonstrated a prototype breath sensor for acetone detection, the Keto-whistle, as a non-invasive device for type-I diabetes monitoring. Hopefully, one or more of our sensors can be used in real life and that, would certainly be an achievement that I will be most proud of.

**5. You have studied in Hefei University of Technology, University of Science and Technology of China and Australian National University. How have these experiences shaped you?**

Prof. Lan Fu: My undergraduate degree in Hefei University of Technology (HUT) is on Applied Physics, specialized in Microelectronics and Optoelectronic, through which I developed my basic knowledge in physics and engineering. I guess my passion for semiconductor devices was also born from there. During my undergraduate years, I also had the opportunity to develop leadership skills by engaging with many student led activities, in particular personal and communication skills. When I did my Masters in University of Science and Technology of China (USTC), I was very lucky to be supervised by Prof. Yuheng Zhang, a well-known expert on high T_c_ superconductors and an academician of Chinese Academy of Science, who trained me the basic research skills through a theoretical project. The valuable research experience and the first two papers published from my master project helped me obtain a full scholarship to do PhD at the Department of Electronic Materials Engineering, Research School of Physics, Australian National University (ANU). In the first few weeks when I just joined the department, I was offered the opportunity to spend some time with each research group of the department to choose my own PhD project. I remember that one day one of the postdocs of Prof. Jagadish showed me an InGaAs/GaAs quantum well laser that he was testing on the day. I saw through an infrared viewer a bright infrared light emitting from a tiny semiconductor laser. This fascinated me so much and I immediately decided that I would work on a project to make semiconductor optoelectronic devices such as lasers. This is indeed one of the most important moments that defined my career.

**Figure:**
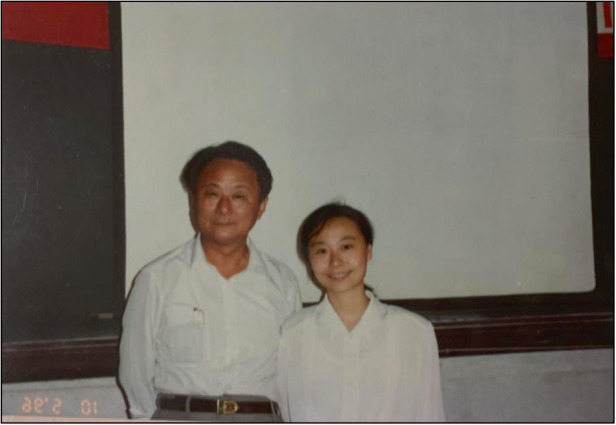
A photo of Professor Lan Fu and her supervisor Prof. Yuheng Zhang when she graduated from her master’s degree at USTC

**6. Chennupati Jagadish, President of the Australian Academy of Sciences and Distinguished Professor of Electronic Materials Engineering at the Australian National University, is your mentor. How has he influenced you?**

Prof. Lan Fu: Prof. Jagadish is my PhD supervisor and I have been working with him since joining the ANU in 1997 as a PhD student. He is the one who led me into the research field of III-V semiconductors, optoelectronics and nanotechnology that I have been passionate about since my PhD. Prof. Jagadish is a great mentor. While providing incredible support and guidance at different stages of my career in so many different ways, at the same time he has given me the freedom of coming up with my own ideas and making up my own decisions, so that I can grow into an independent researcher. More importantly, by being a role mode, he has taught me how to be a good human being, to always be kind, respectful and generous to others.

**Figure:**
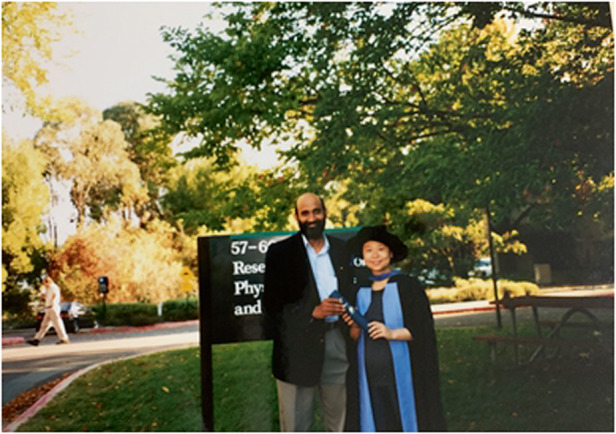
A photo of Professor Lan Fu and her supervisor Prof. Chennupati Jagadish at the time of her PhD graduation

**7. You have won the Australian Research Council (ARC) Future Fellowship award, the IEEE Photonics Society Distinguished Lecturer Award and other honors. As a doctoral supervisor, you have also trained many students. What abilities do you most want to see in your students? What are your expectations for them?**

Prof. Lan Fu: Many people feel that doing a PhD is about developing of very specialized technical knowledge and skills. This is not completely true. There are so many other great skills that one can build through doing a PhD, such as critical thinking, problem solving, writing and oral communication skills, as well as interpersonal and time management skills. Through the PhD training, I certainly want to see my students gradually demonstrate the ability to come up with new research ideas and become more and more independent, to be able to manage the progress and take the full ownership of their research project. More importantly, I expect them to build a set of skills that make them feel confident and prepared to find a job, no matter in or outside academia, and be successful in their future career.

**Figure:**
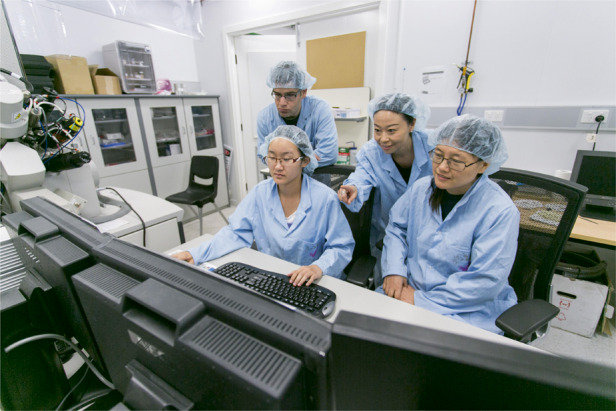
Professor Lan Fu is guiding the students in their work

**Figure:**
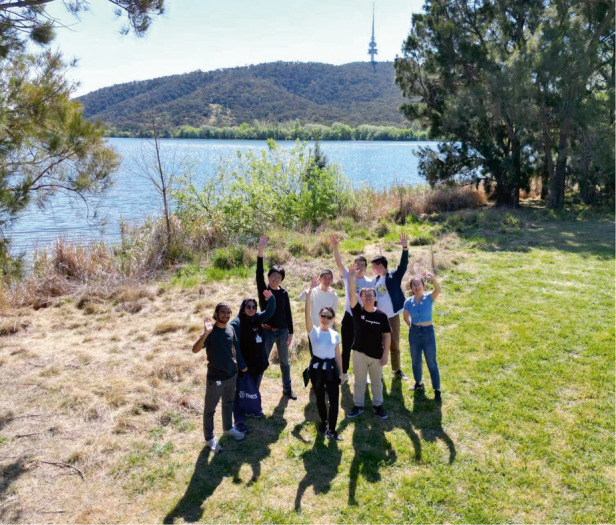
Professor Lan Fu and her team enjoying a lunch time walk

**Figure:**
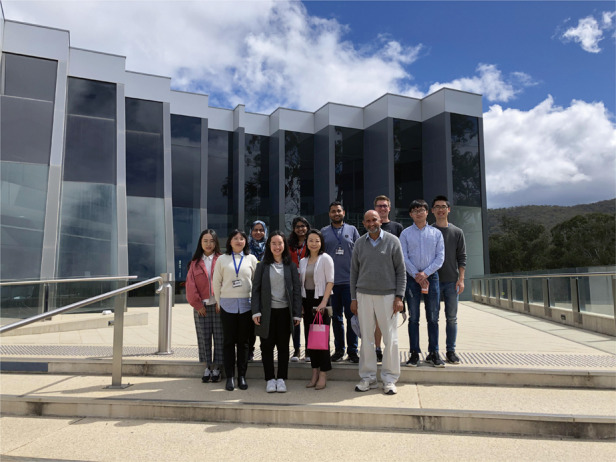
A photo of Professor Lan Fu with Professsor Jagadish and the students

**8. So far, you have published more than 150 SCI papers, edited 5 special issues of journals or conference proceedings. In addition, you are also serving as editorial board member of IEEE Photonics Journal and Opto-Electronic Advances. What advice or suggestions do you have for young researchers on academic paper writing?**

Prof. Lan Fu: My advice would be - read more, think more and write more. The same as many other things, academic writing needs a lot of practice, but the most important thing is to be sure that you really understand your results and be able to explain them clearly. You should present your research data and organize them in a meaningful and logical way. You should have a good knowledge of the relevant literature to your work, and use them effectively to substantiate your discussions. When there are things that you do not understand, do not pretend that they do not exist. Clarify them as much as you can, but be honest if you cannot and suggest possible approaches to address them in the future. Do not rush for publication, spend time polishing your manuscript and ask your colleagues to read and provide feedbacks. It is important to make sure that you prepare your manuscript to the best of your satisfaction before submission. For young researcher, it is extremely important to develop a rigorous and responsible attitude on research and academic writing.

**9. You have been involved with organising of many conferences such as the American Materials Research Society (MRS), Conference on Lasers and Electro-Optics (CLEO), The Optical Society of America (OPTICA), IUMRS and IEEE Photonics Conference, etc. What have you gained and learned from organising these conferences? What do you think makes a good academic presentation?**

Prof. Lan Fu: For a researcher, we benefit a lot from participation of International conferences to disseminate your research, learn about the progress of the field, interact and networking with colleagues. But as you may know, most of the international conferences are run by researchers who volunteer their own time. In addition to enjoying the benefit of attending the conferences, being involved with various societies and organizations of conferences provide me opportunities to interact with colleagues with different research background to gain broader perspectives of the research field. It also allows me to learn different organization approaches, processes and ideas. As to academic presentation, I have listened to many great presentations. There are long ones and short ones, given by both high profile researchers, and fresh PhD students. They all have one thing in common, that is, a good “story” that everyone can learn something from. A good academic presentation should involve general introduction of the research background, a clear explanation of the fundamental science and thorough discussion of the key results. The breadth and depth of these elements should be adjusted and balanced depending on the type of presentation and audience. Of course a clear and nicely presented PPT with eye-catching animations would be very impressive and able to enhance the engagement of the audience. For students, I would stress the importance of practice, in front of different audiences, to get feedbacks, build confidence, and properly manage the time.

**10. In 2022, you participated in the “Blooming Rose in Science” activity sponsored by the Changchun Institute of Optics, Fine Mechanics and Physics (CIOMP), Chinese Academy of Sciences, co-organized by CIOMP together iCANX(Global Technology Innovation Ecological Platform), and served as the host of the second half of the activity. What are your feelings about this activity?**

Prof. Lan Fu: “Blooming Rose in Science” is a great initiative to showcase the wonderful research achievements of female scientists across the world. It features not only well-established professors, but also young researchers who just finished their PhD. In addition to the technical presentations, I also enjoy the short video clips that the presenters shared with us about their families, hobbies and challenges, as well as a lot of wise advice to the students. We all know that in the STEM field women participation is way much lower than the male counterpart. Events like this are extremely important to not only raise the awareness of gender balance, but also provide female role models to encourage bright young female students to pursuit a career in STEM by openly discussing the range of issues, challenges and possible solutions. I really appreciated that CIOMP and iCANX provide such a platform and opportunity for me to contribute to it.

**Figure:**
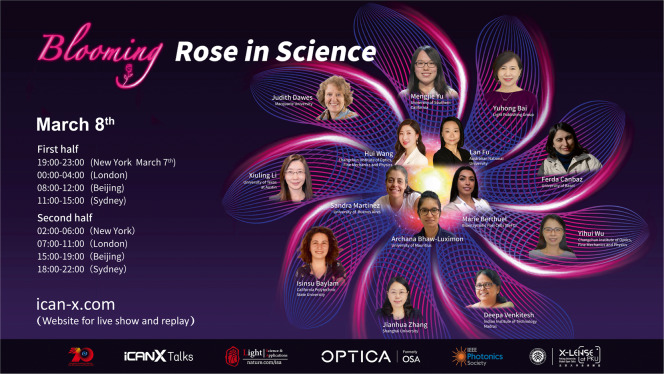
A poster of Rose in Science Event

**Figure:**
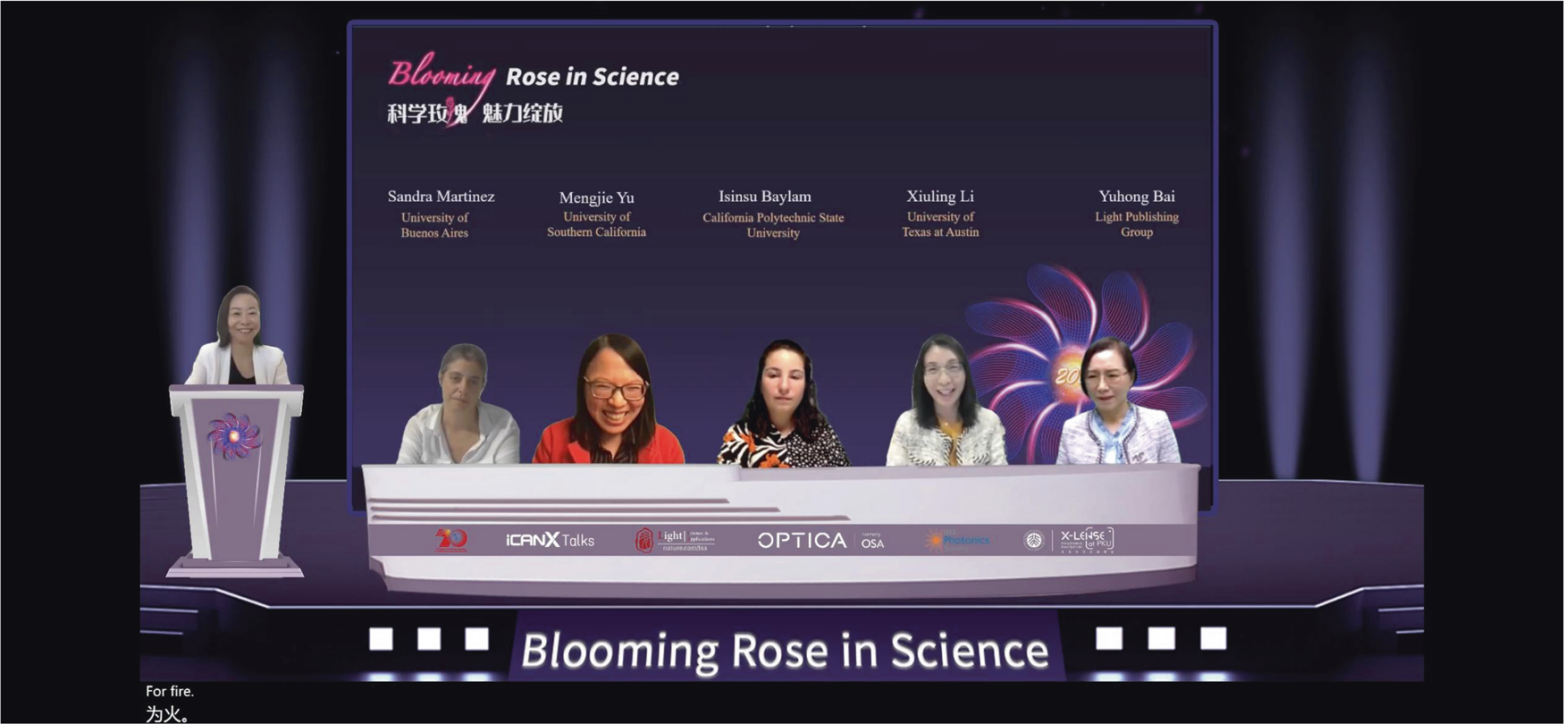
Professor Lan Fu hosted the first half of the Rose in Science Event in 2022

**11. You are now the host of iCANX Talks. Why are you still happy to give your time to this kind of science educational activity in spite of a busy scientific research schedule? How do you balance scientific research, social engagements and personal life?**

Prof. Lan Fu: Since its launch, iCANX Talks have reaches to millions of audiences, including young students, academics and even general publics. It is a wonderful way to promote science, technology and collaboration. For me, it is a privilege to be part of iCANX talks to meet and discuss with so many great scientists from all over the world, and also take this opportunity to give back to the community. Being an academic, research, education and professional service are part of our job. Although we have lots of work to do, we also have some flexibility in arranging our times. To me, the most effective way is to establish a good routine both at work and at home, and keep effective communication with my research team and family. I make sure that I talk to them regularly and be available whenever they need me. I am very fortunate to have a close and supportive family, which make such a balance so much easier.

**12. Do you have any hobbies?**

Prof. Lan Fu: I used to like reading detective stories and watching detective movies. In recent years, ballet, yoga and Pilates become my new hobbies. I enjoy getting up early to do my practice every day. It brings a lot of satisfactions when I make progresses. These exercises also keep me feel energized and more focused throughout the day.

**Figure:**
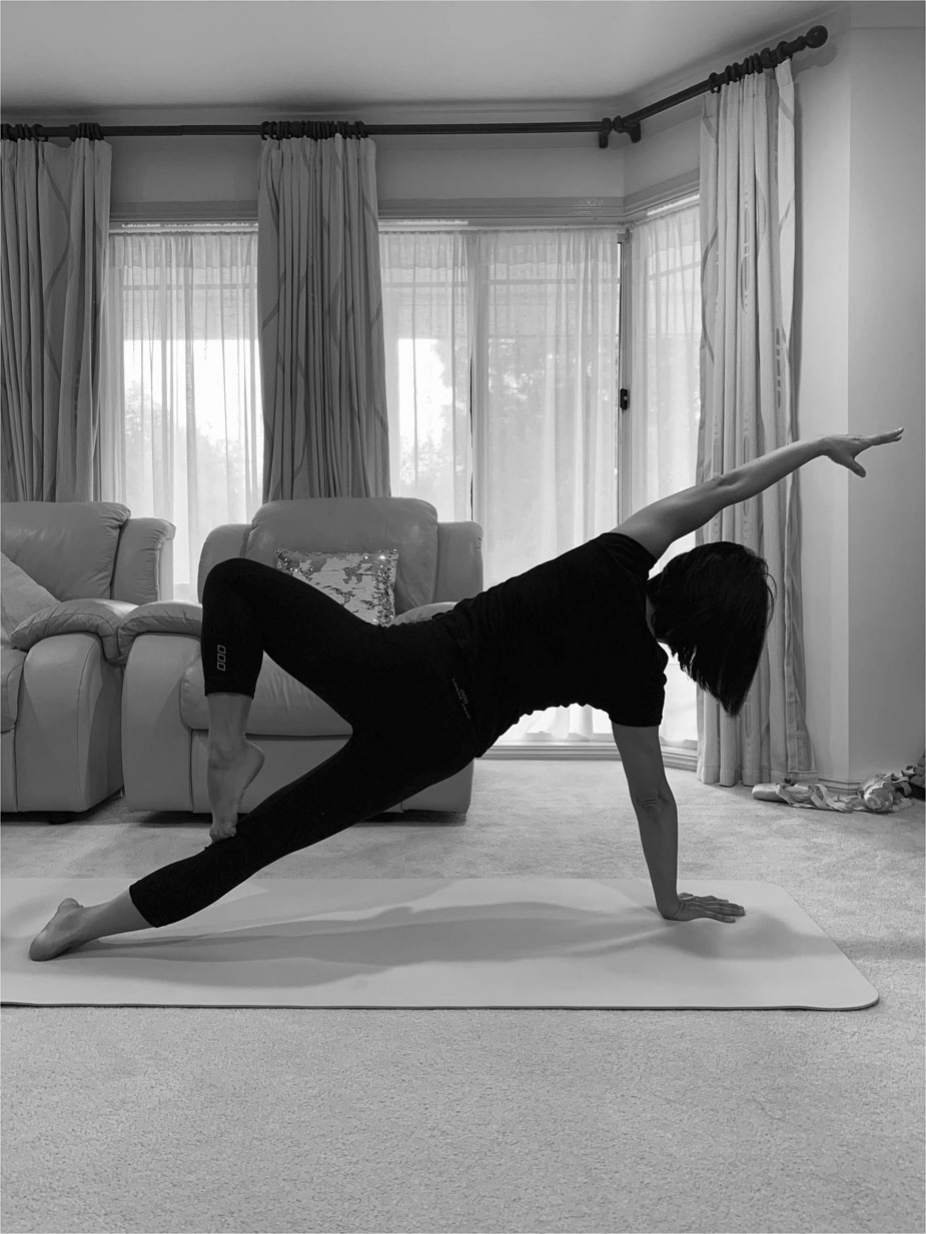
Professor Lan Fu is practicing Pilates

**13. What, in your opinion, are characters a good science worker should have? What advice and expectations do you have for today’s young researchers?**

Prof. Lan Fu: Well, in my opinion, good scientific researchers could exhibit many good characters in many different ways. However there are four characters that I personally value the most important, i.e., integrity, passion, curiosity, and perseverance.

For young researchers - aiming high, but do not rush. Real success takes time to build. Only hard work, good collaboration and perseverance will take you there.

